# Clinical Characteristics of Early-Onset and Late-Onset Multiple Sclerosis in Patients from Lithuania

**DOI:** 10.3390/medicina61010107

**Published:** 2025-01-13

**Authors:** Emilija Šlajūtė, Naglis Vičkačka, Tautvydas Klėgėris, Ingrida Ulozienė, Renata Balnytė

**Affiliations:** 1 Faculty of Medicine, Lithuanian University of Health Science, 44307 Kaunas, Lithuania; 2 Department of Otorhinolaryngology, Faculty of Medicine, Lithuanian University of Health Sciences, 44307 Kaunas, Lithuania; tautvydas.klegeris@lsmu.lt (T.K.); ingrida.uloziene@lsmu.lt (I.U.); 3 Department of Neurology, Faculty of Medicine, Lithuanian University of Health Sciences, 44307 Kaunas, Lithuania

**Keywords:** multiple sclerosis, early-onset multiple sclerosis, late-onset multiple sclerosis, relapse, disability

## Abstract

*Background and Objectives:* Early-onset MS (EOMS) and late-onset MS (LOMS) differ in terms of symptom presentation, disease progression, and disability outcomes. This study aims to evaluate the clinical characteristics of patients with EOMS and LOMS in Lithuania. *Materials and Methods:* A retrospective analysis of patients’ medical records was conducted at the Lithuanian University of Health Sciences, Kaunas Clinics Department of Neurology. This study included 97 patients with multiple sclerosis, of which 34 were diagnosed with EOMS and 63 with LOMS. *Results:* The female/male ratio did not differ significantly in the EOMS group (1.26:1), while in the LOMS group, the female-to-male ratio was 2:1. All EOMS patients were diagnosed with relapsing–remitting multiple sclerosis (RRMS), while in the LOMS group, RRMS was observed in 55.6%, secondary progressive multiple sclerosis (SPMS) was observed in 27%, and primary progressive multiple sclerosis (PPMS) was observed in 17.4% of patients (*p* < 0.001). The most common initial symptoms in the EOMS group were brainstem dysfunction (50%), and sensory (38.2%) and visual (26.5%) disorders, whereas LOMS patients predominantly experienced brainstem dysfunction (50.8%) and motor impairments (47.6%). The EOMS group experienced more clinical relapses in the first year after diagnosis, along with more frequent radiological signs of disease activity compared to LOMS (*p* < 0.001). Both groups demonstrated a significant increase in Expanded Disability Status Scale (EDSS) score at the last follow-up visit compared to the baseline, while the LOMS group had higher EDSS scores both at the baseline and at the last follow-up compared to the EOMS group (*p* < 0.001). Only LOMS patients had an increase in Multiple Sclerosis Severity Score (MSSS) at the last follow-up compared to the baseline (*p* = 0.028), and MSSS was higher than in EOMS patients both at the baseline (*p* = 0.004) and the last follow-up (*p* < 0.001). *Conclusions:* There was no significant gender difference in the EOMS group, whereas in the LOMS group, females were predominant. Both groups had RRMS as the most common disease course. At the onset of MS, brainstem dysfunction was the most common symptom in both patient groups. EOMS patients had a more active disease course, in contrast to LOMS patients, who exhibited higher levels of disability, suggesting a progressive disease.

## 1. Introduction

Multiple sclerosis (MS) is a chronic inflammatory disease characterized by demyelinating and neurodegenerative changes in the central nervous system (CNS) resulting from an autoimmune response triggered by genetic and environmental factors [1]. MS has a variable clinical course consisting of episodes of remission and relapse, and progression of disability. The severity of symptoms depends on the localization and quantity of the focal demyelinated lesions. Symptoms include visual, motor, sensory, cerebellar, or cognitive impairment [2,3].

MS usually starts in patients aged 20–40 years (adult-onset MS), but rare cases are diagnosed when patients develop their first symptoms before the age of 18 years (early-onset MS) or at the age of 50 years or older (late-onset MS). Early-onset MS (EOMS) is diagnosed in 3–10% of all cases of the disease, while late-onset MS (LOMS) is diagnosed in an average of 4.5% of cases [4,5].

The incidence of EOMS and LOMS has been increasing in recent years. It is believed that one of the reasons is the improved accessibility of magnetic resonance imaging (MRI) and the updated diagnostic criteria for MRI of the brain and spinal cord. These advancements allow for more accurate differentiation between vascular, demyelinating, and nonspecific CNS lesions [6]. Although EOMS and LOMS share similarities with adult-onset MS, these forms of the disease have different demographic, clinical, pathophysiological, and prognostic characteristics, resulting in differences in the management tactics, therapeutic approach, and social care needs of the two groups of patients [7].

The pathogenesis and clinical course of EOMS and LOMS may differ due to certain causes of development or clinical expression. Although EOMS and adult-onset MS are fundamentally the same disease, they may present distinct inflammatory profiles due to the developing immune system in children [8]. The relapsing–remitting multiple sclerosis (RRMS) is the initial predominant clinical variant of EOMS, comprising up to 98% of cases [9]. In contrast, LOMS is typically characterized by primary progressive multiple sclerosis (PPMS). This is attributed to the decline in the regenerative properties of microglial cells, macrophages, and T lymphocytes with advancing age, as well as a reduction in brain plasticity, which collectively contribute to the progression of disability [10,11].

As the incidence of both EOMS and LOMS is increasing, it is important to be aware of its clinical features, disease trends, prognostic features, and challenges of disease management to ensure timely diagnosis of MS, early and tailored disease-modifying treatment, and high-quality case management.

## 2. Materials and Methods

### 2.1. Study Design and Data

This study was a retrospective analysis of MS patients at the Department of Neurology in the Hospital of Lithuanian University of Health Sciences (LUHS) Kaunas Clinics. The study period spanned from January 2023 to January 2024. Ethical approval for the study was obtained from the LUHS Department of Bioethics on 19 January 2023. In all, 97 patients diagnosed with EOMS (*n* = 34) and LOMS (*n* = 63) were randomly selected for the study and were classified into corresponding groups. Patient data were retrieved from outpatient medical records and included demographic information (age, sex); clinical information (date of first symptoms and MS diagnosis; the initial symptoms of the disease; clinical course; number of relapses within one year of the diagnosis); MRI findings of the brain and spinal cord (localization and activity of lesions at the time of diagnosis); radiological signs of disease activity (new active, new inactive, or enlarged previous lesions) within the first year of diagnosis); and the Expanded Disability Status Scale (EDSS) score.

We used the results of brain and spinal cord MRI conducted at the time of diagnosis and during the first year after the diagnosis to compare the radiological signs of disease activity between both groups of patients. New active and inactive foci of demyelination and previously detected enlarged foci were considered signs of radiological relapse of the disease. Only 24 EOMS patients and 29 LOMS patients were included in the analysis of radiological relapses during the first year after diagnosis, as not all patients’ data were available.

Disease severity and the patient’s disability were evaluated using EDSS and Multiple Sclerosis Severity Score (MSSS) results taken during remission phases from the first post-diagnosis visit (regarded as a baseline) and the most recent clinic visit. Patients were excluded from disability and disease severity analysis if patients did not visit the Department of Neurology in LUHS Kaunas Clinics during the first year or longer after the diagnosis or less than a year had passed since the diagnosis. In all, the evaluation of EDSS and MSSS included 32 EOMS and 53 LOMS patients at the baseline, and 32 EOMS and 56 LOMS patients with MSSS at the last visit. For an accurate comparison of disability between the patient groups, we measured the severity of the disease according to the MSSS, which was determined based on the patient’s EDSS at the baseline and the last visit, and the duration of disease at the time of evaluation. MSSS was obtained using onset-specific global MSSS matrices constructed using MSBase registry data, and the score was assessed based on six categories, from H1 (mild MS) to H6 (aggressive MS), according to Herbert’s severity grading [12]. According to the MSSS score, disease severity of up to 1.7 indicates mild MS (H1), from 1.7 to 3.4—moderate MS (H2), from 3.4 to 5.0—intermediate IS (H3), from 5.0 to 6.7—accelerated IS (H4), from 6.7 to 8.3—advanced IS (H5), and more than 8.3—aggressive IS (H6) [13].

### 2.2. Statistical Analysis

IBM SPSS Statistics 29.0.1.0. was used for statistical analysis of the data. The distribution of the data was assessed using the Kolmogorov–Smirnov and Shapiro–Wilk criteria. The means of quantitative variables distributed according to Gaussian criteria were compared using the Student *t*-test. If the data were not normally distributed, the Mann–Whitney U test was used to compare two independent samples, and the non-parametric Wilcoxon test was used to compare dependent samples. For the statistical analysis of qualitative attributes, the non-parametric Chi-square (χ^2^) criterion for independence of attributes was used. Differences between attributes were considered statistically significant if the calculated significance level (*p*-value) was lower than the chosen significance level (α = 0.05).

## 3. Results

Out of 97 patients included in this study, 34 patients (35.1%) were diagnosed with EOMS and 63 (64.9%) with LOMS. The female/male ratio did not differ significantly in the EOMS group (1.26:1) (*p* > 0.05), while in the LOMS group, two-thirds of patients were female (female-to-male ratio—2:1) (*p* = 0.008). In the EOMS group, the average age of the patients at the onset of the disease was 14.94 (SD = 1.8), and, at the time of diagnosis, it was 16.29 (SD = 2.7). At the onset of the disease, there were more children older than 12 years (*n* = 30; 88.24%) than children under 12 (*n* = 4; 11.76%) (*p* < 0.01). In the LOMS group, the mean age of patients at the onset of the disease was 53.63 (SD = 4.1) and 55.81 (SD = 4.4) at the time of diagnosis. The median duration of follow-up for EOMS patients was 10.5 (1–31) years, and 5 (1–22) years for LOMS patients (*p* = 0.001).

All EOMS patients were diagnosed with RRMS (*n* = 34; 100%), while in the LOMS group, RRMS was observed in 55.6% (*n* = 35) of patients and SPMS in 27% (*n* = 17), while PPMS was the least observed course of disease (*n* = 11; 17.4%) (*p* < 0.001). The average time for LOMS patients to transition from RRMS to SPMS disease course was 7.06 years (SD = 3.19).

Unifocal symptoms at disease onset were more common in EOMS patients (*n* = 21; 61.8% vs. *n* = 18, 28.6%), while multifocal symptoms were more common among LOMS patients (*n* = 45, 71.4% vs. *n* = 13, 38.2%) (*p* = 0.001). At the beginning of the disease in EOMS patients, the most frequent symptoms were signs of brainstem dysfunction (*n* = 17; 50%), followed by sensory disorders (*n* = 13; 38.2%) and visual disorders (*n* = 9; 26.5%). Among all symptoms in the LOMS group, brainstem dysfunction (*n* = 32; 50.8%) and motor dysfunction (*n* = 30; 47.6%) were the most observed, and the latter was statistically significantly more frequent in LOMS patients than EOMS (*p* = 0.004). Pelvic dysfunction, cognitive dysfunction, and fatigue were significantly less prevalent than other symptoms in both patient groups (*p* < 0.001); however, pelvic dysfunction was statistically more common in the LOMS group (*p* = 0.044) (Table 1).

Table 2 presents the localization and activity of demyelinating lesions on the initial MRI of the brain. The most common sites of lesions were periventricular and, in the corpus callosum, both in EOMS (*p* = 0.014) and LOMS (*p* = 0.39) patients. While frequencies of active and inactive lesions were equal in EOMS patients, inactive demyelinating lesions were strongly characteristic of LOMS (*n* = 53; 84.1%) (*p* < 0.001) and significantly more frequent than in EOMS patients (*p* < 0.001).

EOMS patients experienced more clinical relapses within the first year after diagnosis than LOMS patients (*p* = 0.026) and, significantly more often, had three relapses in the first year (*p* = 0.006), but there was no significant difference in number of patients with at least one relapse and no relapses in the first year of diagnosis among the groups (*p* > 0.05). Patients who experienced relapses were more likely to experience only one relapse in the first year of diagnosis, both in EOMS (*p* = 0.032) and LOMS (*p* < 0.001) groups.

In the first year of diagnosis, the brain and spinal cord MRI revealed signs of disease activity in a high proportion of EOMS patients (*n* = 21; 87.5%) (*p* < 0.001), whereas in LOMS patients, new or enlarged lesions more often were undetected (*n* = 18; 62.1%), but this was not statistically significant (*p* > 0.05). All combined signs of radiological disease activity (new active, new inactive, or enlarged former lesions) in the first year of diagnosis were more common in EOMS patients compared to LOMS patients (*p* < 0.001). New active demyelinating lesions were a rare finding in the LOMS group (*n* = 1; 3.4%) (*p* < 0.001) and were more characteristic of EOMS patients compared to LOMS patients (*p* < 0.001) (Table 3).

**Figure 1:**
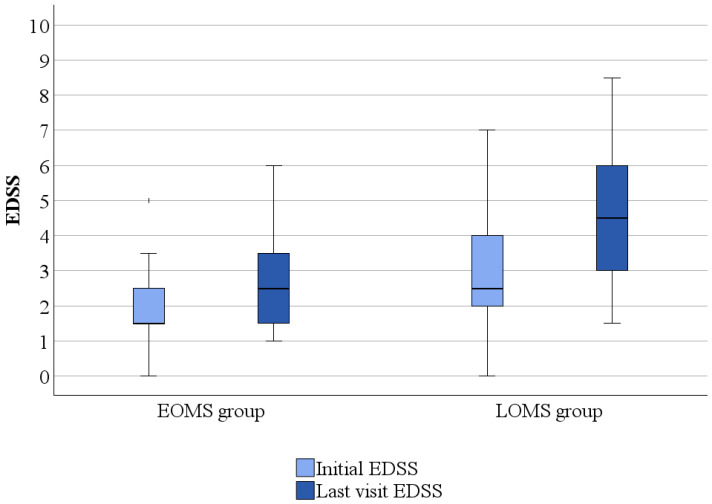
Comparison of Expanded Disability Status Scale (EDSS) between early-onset and late-onset multiple sclerosis patients.

The median EDSS at the baseline for EOMS patients was 1.5 (range of 0–5) and 2.5 (range of 1–6) at the last visit. EDSS at the last visit was higher than baseline EDSS in the EOMS group (*p* = 0.013). The median EDSS at the baseline for LOMS patients was 2.5 (range of 0–7) and 4.5 (range of 1.5–8.5) at the last visit. EDSS was higher at the last visit compared to the baseline in the LOMS group (*p* < 0.001). EDSS at the last visit and baseline EDSS were higher in the LOMS group compared to the EOMS group (*p* < 0.001) (Figure 1).

**Figure 2:**
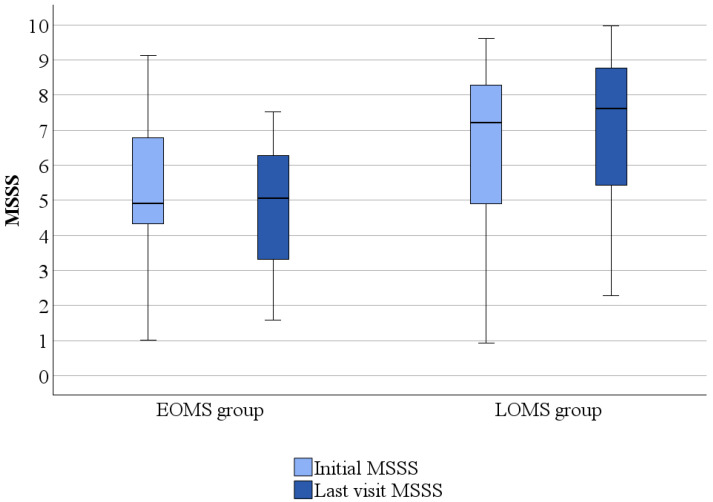
Comparison of Multiple Sclerosis Severity Score (MSSS) in early-onset and late-onset multiple sclerosis patients.

The median MSSS at the baseline for EOMS patients was 4.9 (1.02–9.13) (representing moderate MS, H3 according to Herbert’s severity grading) and 5.05 (1.59–7.53) (representing accelerated MS, H4 grade according to Herbert’s severity grading) at the last visit. There was no significant difference between baseline MSSS and MSSS at the last visit in the EOMS group (*p* = 0.168). The median MSSS at the baseline in LOMS patients was 7.2 (0.93–9.31) (advanced MS, grade H5) and 7.61 (2.28–9.97) (advanced MS, grade H5) at the last visit. MSSS was higher at the last visit compared to the baseline in the LOMS group (*p* = 0.028). The LOMS group had a significantly higher MSSS Severity Score at the baseline (*p* = 0.004) and at the last visit (*p* < 0.001) than the EOMS group (Figure 2).

## 4. Discussion

Early-onset MS and late-onset MS are rare forms of the disease; hence, not many studies have compared these patient groups to date. It is well known that both EOMS and LOMS are more common in women than in men, and a female-to-male ratio ranges from 2.8:1 to 3.88:1 and 1.4:1 to 2.4:1 in both groups, respectively [14,15,16,17]. However, in our study, there was no significant difference in the ratio between females and males in the EOMS, while the majority of LOMS patients were women. Supposedly, the development and onset of MS in girls during adolescence are related to sex hormones and the onset of menarche [18]. Sex hormones may also induce LOMS development in women; however, there are no reliable studies to date to confirm a possible link between menopause and the risk of MS. Nevertheless, menopause is associated with faster disability progression and reduced disease activity in MS patients due to accelerated neurodegenerative processes and impaired regenerative mechanisms of the CNS, which may be due to decreased estrogen levels [19,20,21].

Hormonal changes and reduced regenerative capabilities of the brain associated with age explain the tendency of LOMS to develop as a PPMS at the onset of the disease, while EOMS in, on average, 98.6% of cases begins with an RRMS, reflecting high disease activity at a young age [14,16]. In our study, RRMS was observed in all EOMS patients and was also dominant among LOMS patients (55.6% of cases), but, comparing both groups, RRMS was more typical of EOMS. The PPMS was the least frequent clinical course in our patients with LOMS (17.4% of patients). In studies conducted in different countries, the frequency of LOMS patients with a PPMS ranges from 16 to 83%, while, according to the data of separate meta-analyses, the RRMS occurs in 50–58% of LOMS patients [14,22,23,24,25].

We found that at the beginning of the disease, brainstem dysfunction was the most common symptom in EOMS patients. According to the literature, brainstem dysfunction is commonly found in MS patients under the age of 12 years. However, there were only a few children under 12 in our EOMS group, suggesting that these disorders may also occur in older children [26]. In most studies, visual disturbances were the most common finding in pediatric patients, while, in our study, it was the third most frequent symptom [15,27].

According to other studies, the most characteristic symptom in LOMS patients is motor function impairment, usually caused by foci of demyelination in the spinal cord [14,25,28]. In this study, brainstem dysfunction and impairment of motor function were the most common signs of the disease in LOMS patients.

There was no difference in the frequency of active and non-active lesions among our EOMS patients at the time of diagnosis, while other studies proved that active gadolinium-enhancing lesions are widely usual. Gadolinium accumulation is a temporary phenomenon lasting up to 3 weeks; therefore, MRI does not always help to detect signs of disease activity [29]. Active lesions were found in only 15% of LOMS patients, which was statistically significantly less than in EOMS patients, indicating a difference in disease activity between these forms of MS. Studies with similar results estimated that active inflammatory demyelination rarely occurs in LOMS and a decrease in neuronal density is more common, which is associated with more rapidly progressive disease [30,31].

A total of 64.7% of EOMS patients experienced at least one clinical relapse, and 87.5% of EOMS patients had signs of disease activity on MRI in the first year of diagnosis. These results align with the results of other studies, in which up to 80% of EOMS patients experienced at least one clinical relapse, and new radiological signs of disease activity were detected in about 40–60% of cases within the first year of diagnosis, confirming that MS at a young age has high activity at the beginning of the disease [26,32,33,34]. Even though there was no statistical significance regarding the frequency of radiological and clinical relapses among our LOMS patients, they had fewer radiological and clinical relapses when compared to EOMS patients, indicating that LOMS is characterized by a less aggressive course of the disease.

At the baseline, EOMS patients had a median EDSS of 1, indicating slow progression of MS at disease onset. The results correspond to other studies, where median EDSS ranges from 1 to 1.5 [33,35,36]. The median EDSS in LOMS patients was 2.5 at the baseline and 4.5 at the last visit in our study, with the latter being lower compared to other studies, where the median EDSS score was mostly 6.5, regardless of clinical course [5,24]. Although the median follow-up time of LOMS patients did not significantly differ between the studies, possible reasons for the difference in the EDSS score could be differences in disease duration and proportions between subjects with RRMS and progressive disease courses.

At the baseline, EOMS patients had a median MSSS score of 4.9, representing MS of intermediate severity (according to Herbert’s severity grading). In this study, EOMS patients had more severe disease compared to other studies in which disease severity at the beginning of the disease averaged up to 3.4 points (moderate disease), according to the MSSS [37]. The median MSSS in LOMS patients was 7.2 at the baseline and 7.61 at the last visit, when MSSS ranging from 6.7 to 8.3 corresponds to the advanced MS. There are very few studies evaluating disease severity, according to the MSSS, in patients with LOMS. Compared to a large longitudinal study conducted in the United States, in which LOMS patients had a median MSSS of 2.5 (mild MS) and 4.8 (moderate MS) at the last visit, in our study, LOMS patients were characterized by more severe disease [38].

In the present study, we found EDSS and MSSS scores to be significantly higher at the time of the diagnosis and the last visit in individuals with LOMS, reflecting previous studies [15]. These findings suggest that LOMS, due to age-related insufficient regenerative properties of the CNS and more rapid neurodegeneration, is characterized by more progressive disability than EOMS. The reason for the higher EDSS in patients with LOMS, and consequently the corresponding MSSS, may also be the higher frequency of motor impairments, which may lead to higher disability scores [30].

This is the first study of the Lithuanian population that aims to evaluate the clinical characteristics of LOMS patients and compare them to EOMS patients. This research improves the understanding of clinical features and trends in disease activity and progression in EOMS and LOMS patients, helping to determine the appropriate disease management. However, this study is limited by the small sample of both patient groups used in the analysis. Also, there was a lack of data on MRI performed during the first year after the diagnosis; consequently, the comparison of radiological relapses in the first year is restricted by a small sample. Moreover, this is a retrospective study, and the median follow-up time of both patient groups differs; a comparison of EDSS scores may be inaccurate. For accurate evaluation and comparison of disability in patients, we used MSSS scores to eliminate inaccuracies caused by the duration of observation.

## 5. Conclusions

In the EOMS group, there was no significant difference in frequency between sexes, while, in the LOMS group, a larger portion of patients were women. RRMS was the most common disease course in both groups of patients, but it was more characteristic of EOMS. At the onset of MS, brainstem dysfunction was the most common symptom in both patient groups, but the EOMS group was also present with sensory and visual impairments, while LOMS patients were characterized with motor dysfunctions, which occurred significantly more often in LOMS compared to EOMS. Multifocal symptoms were more characteristic of LOMS patients, while mono-focal symptoms were more characteristic of EOMS patients. EOMS patients were characterized by a more active disease, in contrast to the LOMS group, as active demyelinating lesions at the time of diagnosis and during the first year of diagnosis, as well as clinical relapses and all radiological signs of disease activity during the first year of diagnosis, were more often observed. In both groups, disability, according to the EDSS, was significantly higher on the last visit than on the first visit after diagnosis, while disease severity, according to the MSSS, increased only in the LOMS group. LOMS patients had higher EDSS and MSSS scores at the baseline and the last visit compared to the EOMS patients, suggesting a progressive form of the disease.

## Figures and Tables

**Table 1:** Initial symptoms of multiple sclerosis in early-onset and late-onset multiple sclerosis patients.

Clinical Symptoms at the Onset	Patient Groups		
	EOMS * Group *n*, (%)	LOMS * Group *n*, (%)	*p*-Value
Monofocal	21 (61.8)	18 (28.6)	*p* = 0.001
Multifocal	13 (38.2)	45 (71.4)	*p* = 0.001
Visual	9 (26.5)	11 (17.5)	NS *
Sensory	13 (38.2)	29 (46)	NS *
Motor	6 (17.6)	30 (47.6)	*p* = 0.004
Brainstem dysfunction	17 (50)	32 (50.8)	NS *
Cerebellar dysfunction	7 (20.6)	24 (38.1)	NS *
Pelvic dysfunction	0	7 (11.1)	*p* = 0.044
Pain	4 (11.8)	18 (28.6)	NS *
Cognitive dysfunction	1 (2.9)	6 (9.5)	NS *
Fatigue	2 (5.9)	7 (11.1)	NS *

* EOMS—early-onset multiple sclerosis, LOMS—late-onset multiple sclerosis, NS—not significant.

**Table 2:** Magnetic resonance imaging characteristics of early-onset and late-onset multiple sclerosis patients at the time of diagnosis.

Initial MRI * Characteristics	Patient Groups		
	EOMS * Group *n*, (%)	LOMS * Group *n*, (%)	*p*-Value
Localization of demyelinating foci			
Periventricular, *n* (%)	29 (85.3)	44 (69.8)	NS *
Subcortical, *n* (%)	19 (55.9)	24 (38.1)	NS *
Corpus callosum, *n* (%)	24 (70.6)	39 (61.9)	NS *
Brainstem, *n* (%)	11 (32.4)	33 (52.4)	NS *
Cerebellum, *n* (%)	14 (41.2)	25 (39.7)	NS *
Spinal cord, *n* (%)	12 (35.3)	24 (38.1)	NS *
Activity of demyelinating foci			
Inactive foci, *n* (%)	17 (50)	53 (84.1)	*p* < 0.001
Active foci, *n* (%)	17 (50)	10 (15.9)	*p* < 0.001

* MRI—magnetic resonance imaging, EOMS—early-onset multiple sclerosis, LOMS—late-onset multiple sclerosis, NS—not significant.

**Table 3:** Signs of disease activity in the first year of diagnosis of early-onset and late-onset multiple sclerosis patients.

Signs of Disease Activity in the First Year of Diagnosis		Patient Groups			
		EOMS * Group *n*, (%)	LOMS * Group *n*, (%)	*p*-Value	
Clinical relapses					
Median number of relapses		1 (0–3)	0 (0–3)	*p* = 0.026	
Number of patients with at least one relapse		22 (64.7)	30 (47.6)	NS *	
Number of relapses	1	12 (54.5)	22 (73.3)	NS *	
	2	2 (9.1)	7 (23.3)	NS *	
	3	8 (36.4)	1 (3.3)	*p* = 0.006	
Radiological relapses					
Signs of radiological relapses	New active foci	12 (50)	1 (3.4)	*p* < 0.001	*p* < 0.001
	New inactive foci/previously detected enlarged foci	9 (37.5)	10 (34.5)	NS *	
New foci undetected (no relapse)		3 (12.5)	18 (62.1)	*p* < 0.001	

* EOMS—early-onset multiple sclerosis, LOMS—late-onset multiple sclerosis, NS—not significant.

## Data Availability

The original contributions presented in this study are included in the article. Further inquiries can be directed to the corresponding author.
